# Nutrient-related metabolite profiles explain differences in body composition and size in Nile tilapia (*Oreochromis niloticus)* from different lakes

**DOI:** 10.1038/s41598-021-96326-3

**Published:** 2021-08-19

**Authors:** Tokuma Negisho Bayissa, Michelle Geerardyn, Donna Vanhauteghem, Mulugeta Wakjira, Geert Paul Jules Janssens

**Affiliations:** 1 Department of Nutrition, Genetics and Ethology, Faculty of Veterinary Medicine, Ghent University, Heidestraat 19, 9820 Merelbeke, Belgium; 2 Department of Biology, College of Natural Sciences, Jimma University, P.O. Box 378, Jimma, Ethiopia

**Keywords:** Biochemistry, Zoology, Ecology, Environmental sciences

## Abstract

This study investigated how metabolite analysis can explain differences in tissue composition and size in fish from different habitats. We, therefore, studied Nile tilapia (*Oreochromis niloticus*) from three Ethiopian lakes (Gilgel Gibe, Ziway, and Langano) using dried bloodspot (DBS) analysis of carnitine esters and free amino acids. A total of sixty (N = 60) Nile tilapia samples were collected comprising twenty (n = 20) fish from each lake. The proximate composition of the targeted tissues (muscle, skin, gill, gut, and liver) were analyzed. The DBS samples were analyzed for acylcarnitine and free amino acid profiles using quantitative electrospray tandem mass spectrometry. Metabolite ratios were calculated from relevant biochemical pathways that could identify relative changes in nutrient metabolism. The mean weight of Nile tilapia sampled from each lake showed weight variation among the lakes, fish from Lake Ziway were largest (178 g), followed by Gilgel Gibe reservoir (134 g) and Lake Langano (118 g). Fish from Gilgel Gibe showed significantly higher fat composition in all tissues (*P* < 0.05) except the liver in which no significant variation was observed. The source of fish affected the tissue fat composition. Marked differences were observed in Nile tilapia metabolic activity between the lakes. For instance, the lower body weight and condition of the fish in Lake Langano coincided with several metabolite ratios pointing to a low flow of glucogenic substrate to the citric acid cycle. The low propionyl to acetylcarnitine ratio (C3:C2) in Gilgel Gibe fish is indicating that more of the available acetyl CoA is not led into the citric acid cycle, but instead will be used for fat synthesis. The metabolic markers for lipogenesis and metabolic rate could explain the high-fat concentration in several parts of the body composition of fish from Gilgel Gibe. Our results show that nutrition-related blood metabolite ratios are useful to understand the underlying metabolic events leading to the habitat-dependent differences in the growth of Nile tilapia, and by extension, other species.

## Introduction

Fish populations are declining worldwide because of several threats^1–3^. The causes are anthropogenic factors: habitat destruction, the introduction of new species, pollution, and overfishing^4–7^ and are accompanied by climate change^8^. These threats not only affect the fish production potential but also the nutritive value and metabolic activities of fish. They are altering the aquatic ecosystem by shifting distributions, nutrient load, and phenologies^9,10^, but understanding how fish metabolically respond to these changes remains a challenge^11^.

Reports indicate that fish can perform differently in different ecosystems: even the same fish species in different habitats show variation in proximate composition especially on fat content^12,13^. This may be directly linked with the composition of the food web in each habitat^14^. To understand how the food web affects fish growth, there is a need to identify how nutrition is affecting fish metabolism. Environmental conditions have been reported as key factors to affect fish metabolism^15^, yet studies on fish metabolism are usually performed under laboratory conditions, minimizing the complexity that is present in nature^16–18^. In such experiments, fish blood analyses have been used as a tool to investigate amino acid and fatty acid catabolism^16–20^.

Most of these studies were conducted under controlled parameters like temperature, oxygen, and given a known diet. For instance, in carp, mildly elevated temperature resulted in increased amino acid catabolism^16^ and dietary L-carnitine supplementation stimulated lipid catabolism and increased glycogen and protein deposition in Nile tilapia muscle^21^. However, fish in their natural ecosystem are experiencing a heterogeneous habitat and complex flow field^22,23^. A natural ecosystem harbors a diversity of factors, making it hard to identify the most limiting factors for fish growth without unraveling the effects on metabolism mechanistically.

Despite free ranging fish are challenged by heterogeneous environmental changes, the available organic nutrient from primary and secondary producers in the aquatic environment affect fish growth and body composition. Hence, the available nutrient type that affect fish metabolism are likely to have a direct impact on the fish growth and tissue nutrient composition^24^. In plasma and in whole blood, total carnitine is present in the form of either free carnitine (non-esterified molecule), or acylcarnitines (esterified form). The ratio of acylcarnitine to free carnitine is a biomarker to measure the acylated carnitines versus the free carnitines. Measuring tissue concentration of carnitine is crucial to get insight on how an organism copes with the diet available in their environment, because changes in carnitine requirement are correlated with changes in nutritional status^25^.

For decades, analyzing of acylcarnitine profile was limited to the field of human medicine to investigate biochemical screening of disorders of fatty acid oxidation and organic acid metabolism in humans^26^. Recently, the studying of nutrient metabolites via analysis of acylcarnitine profiling using dried blood spot samples has been extending to other animal species including aquatic organisms. Worku et al.^27^ measured seasonal and agro-ecological effects on nutritional status in tropical ranging dairy cows^28^; investigated the real image of nutrient use in anuran (frogs) metabolism.

To our knowledge, no results have been reported on nutrient metabolite profiles to explain differences in fish performance in different wild aquatic ecosystems using acylcarnitines and amino acid profiling. Therefore, we here used dried bloodspot analysis of nutrient metabolite profiling (carnitine esters and free amino acids) to identify why Nile tilapia differs in body composition and size between three different Ethiopian lakes.

## Results

### Physicochemical properties of the three Lakes

The physicochemical properties of the three Ethiopian lakes used in this study were presented in Table 1. The two Rift Valley lakes (Ziway and Langano) showed no observed difference. However, Gilgel Gibe showed notably lower in all parameters except dissolved oxygen and ammonia in which there is no pronounced differences among the three lakes.

**Table 1:** Physicochemical properties of the three Ethiopian lakes in this study.

Lake	T (°C)	DO (mg/L)	EC (μS/cm)	pH	NH_3_ (mg/L)	NO_3_^-^ (mg/L)
Ziway	27.01	6.31	1345	8.55	0.64	7.97
Langano	27.40	6.11	1634	8.73	0.68	9.26
Gilgel Gibe	23.30	6.74	456	7.56	0.07	2.81

(In situ measurement); T = Temperature, DO = Dissolved oxygen, EC = electric conductivity.

### Morphometric feature of Nile tilapia from the three lakes

Morphometric feature of Nile Tilapia caught for this study from the three lakes were presented Table 2. Though, fish sample collection method and material (standard gillnet) used were same, the caught composition showed distinctive weight length variation. Accordingly, the caught Nile tilapia from Lake Ziway were largest (178 g), followed by Gilgel Gibe reservoir (134 g) and Lake Langano (118 g).

**Table 2:** Morphometric features of Nile tilapia caught from three lakes in Ethiopia (mean, pooled SEM, n = 20).

	Gilgel Gibe	Ziway	Langano	*P*	SEM
Weight (g)	134^b^	178^c^	118^a^	< 0.001	6
Length (cm)	19.57^b^	22.44^c^	17.31^a^	< 0.001	0.30
Weight: Length	7.78^b^	7.86^b^	4.70^a^	0.002	0.20

Different superscripts within a row indicate significant differences at *P* < 0.05.

### Crude fat and crude protein composition of Nile tilapia tissues

Great variation was observed in crude fat concentrations among tissues, but only two tissues (gill and gut) showed variation in crude protein composition among the lakes (Table 3). The source of fish affected the composition of crude fat in several tissues. For most tissues, crude fat concentration was highest in Gilgel Gibe fish and lowest in Lake Langano fish. Crude protein concentration was slightly higher in guts from Gilgel Gibe fish and gills from Ziway fish. For the other tissues, there were no substantial differences in crude protein concentration between the lakes.

**Table 3:** Crude fat and crude protein content of Nile tilapia tissues from three lakes in Ethiopia (g/kg on dry matter basis).

Macronutrient	Tissues	Gilgel Gibe	Ziway	Langano	*P*	SEM
Crude fat	Muscle	34^b^	31^ab^	26^a^	0.008	2
	Gill	287^b^	215^a^	219^a^	< 0.001	7
	Skin	49^b^	46^b^	31^a^	< 0.001	3
	Gut	179^b^	169^a^	168^a^	0.011	5
	Liver	165	166	171	0.131	4
Crude protein	Muscle	856	851	844	0.28	8
	Gill	473^ab^	496^b^	445^a^	0.005	14
	Skin	712	690	684	0.061	11
	Gut	407^b^	399^ab^	386^a^	0.004	5
	Liver	604	603	606	0.08	10

Different superscripts within a row indicate significant differences at *P* < 0.05.

### Selected acylcarnitine and free amino acids ratio

The results of selected acylcarnitine and free amino acids ratio are presented in (Table 4). Marked differences were observed in Nile tilapia metabolic activity among the lakes. The C2:C0 ratio, here used as a marker for metabolic rate, was higher in Nile tilapia from Gilgel Gibe than Ziway, with intermediate values for Langano. The C3:C2 ratio was higher in Nile tilapia from Lake Ziway than the two other lakes. In Langano fish, the 3OHC4:C2 ratio was lower than fish from the other lakes. The C4DC: C3 ratio was highest in Gilgel Gibe, followed by Langano and then Ziway. Yet, the C4DC: val + met ratio was lower in Gilgel Gibe than in Langano, with Ziway as intermediate. Langano fish had a lower C3DC: C2 ratio and a lower 3OH-LCFA: LCFA ratio than fish from the other lakes. The ratio of valine to leucine in Gilgel Gibe fish was significantly lower compared to other lakes.

**Table 4:** Ratios of selected acylcarnitines and free amino acids in Nile tilapia blood from the three Ethiopian lakes.

Metabolite ratio (µmol/L)	Gilgel gibe	Zeway	Langano	P	SEM
C2: C0	1.03^b^	0.84^a^	0.98^ab^	0.028	0.2
C3: C2	0.07^a^	0.16^b^	0.08^a^	< 0.001	0.05
3OHC4: C2	0.018^b^	0.017^b^	0.006^a^	< 0.001	0.001
C4DC: C3	0.06^c^	0.02^a^	0.04^b^	< 0.001	0.005
C4DC: Valine methionine	2.1 × 10^-4a^	2.3 × 10^-4ab^	3.2 × 10^-4b^	0.02	0.001
C3DC: C2	0.002	0.002	0.001	0.5	0.001
Valine: Leucine	0.7^a^	0.8^b^	0.8^b^	0.002	0.001
3-OH LCFA:LCFA	0.47^b^	0.49^b^	0.27^a^	0.01	0.02

C2:C0 = acetyl carnitine: free carnitine; C3:C2 = propionyl: acetyl carnitine; 3OH-C4:C2 = 3-hydroxybutyryl: acetyl carnitine; C4DC:C3 = methylmalonyl: propionyl carnitine; C4DC:val + met = methylmalonyl carnitine: (valine + methionine); C3-DC:C2 = malonyl carnitine: acetyl carnitine; val: leu valine: leucine and 3-OH LCFA:LCFA = sum of 3-hydroxy-long-chain fatty acids: sum of long-chain fatty acids. SEM = standard error of the mean.

^a,b,c,^Mean values with different letters within the row are significantly different (*P* < 0.05).

## Discussion

Despite using the same fishing technique in the three lakes, a profound difference in fish size and weight were found. Although other factors may have influenced the fish size of the catch in every lake, fishermen confirmed that it was representative of a typical catch in those lakes. Therefore, it can be concluded that the food webs and conditions in the lakes exerted different growth patterns. This is as such not a novel finding, but we here demonstrated that targeted metabolite analysis can provide a mechanistic explanation for the observed differences in growth performance, as a first step in identifying the nutritional limitations in a particular habitat. The carnitine ester and free amino acid profile have been used in several species including fish to demonstrate differences in nutrient metabolism, for instance^20,21^.

The citric acid cycle, as the most efficient energy-producing pathway in the body, needs substrates for acetyl coenzyme A (CoA) and oxaloacetate to form citric acid. In an aquatic environment, digestible carbohydrates are very limited, meaning that glucose will not be available as the main precursor for oxaloacetate in the citric acid cycle. Instead, to form oxaloacetate, fish will need to use amino acids from dietary protein or—in case of a fair amount of intestinal microbial fermentation—propionic acid. Several metabolite ratios reflecting amino acid and propionic acid use were indeed lowest in Gilgel Gibe fish. First, the low propionyl- to acetylcarnitine ratio (C3:C2) in Gilgel Gibe fish is indicating that more of the available acetyl CoA is not led into the citric acid cycle, but instead will be used for fat synthesis. Although only numerically, the higher ratio of malonyl- to acetylcarnitine is confirming that conclusion, since malonyl CoA is a marker for lipogenesis. Furthermore, prominent glucogenic amino acids as methionine and valine were less converted in methylmalonyl- and succinyl carnitine (witnessed by the C4DC:met + val ratio), leading to less precursor provision for oxaloacetate in the citric acid cycle to link with acetyl CoA (low C4DC: C3 ratio). The final support for the higher fat accretion in Gilgel Gibe fish, is the lower free amino acid ratio of valine to leucine, because valine is a typical glucogenic amino acid (hence precursor for oxaloacetate), whereas leucine is strictly lipogenic and can only provide acetyl CoA.

It thus appears that Gilgel Gibe harbors environmental conditions that lead to a relatively lower supply of glucogenic substrate in the diet, likely because of the relatively lower availability of protein-rich diet items. When considering the acetylcarnitine to free carnitine ratio (C2:C0) as an indicator of the metabolic activity in fish, the higher ratio in Gilgel Gibe fish compared with larger-sized Lake Ziway fish, suggests that Gilgel Gibe still provides sufficient amounts of food for the fish. The trophic status of the lakes indeed differs substantially: for instance, Lake Ziway is characterized as a eutrophic lake^29,30^, and mainly dominated by chroococcal cyanobacteria with numerous Microcystis species^31,32^. Most cyanobacteria including Microcystis species are known to have poor nutritional value and less palatable to the zooplankton (rotifers and crustaceans)^33^, which is the main diet for Nile tilapia^34,35^. In contrast, Lake Langano is characterized as a mesotrophic lake^30^, with lower primary productivity. Gilgel Gibe has a mesotrophic status^36^, but it is a reservoir that could have developed a different food web than the two lakes.

The smaller size of the fish from Lake Langano agrees with a lower metabolic activity (lowest C2:C0 ratio). Besides, the ratio of 3-hydroxy forms of long-chain fatty acids to those long-chain fatty acids is lowest in fish from this lake, showing a low potential of lipogenesis that reflects their low body energy reserves. These fish seem to face a challenge to find sufficient food: despite a high proportion of protein catabolized to provide a glucogenic substrate to the citric acid cycle (high C3:C2, high C4DC: val + met), these metabolites do not sufficiently enter the citric acid cycle as witnessed by the low C4DC: C2 ratio.

The ratio of 3-hydroxybutyryl- to acetylcarnitine (3OHC4:C2) indicates the proportion of acetyl CoA that cannot enter the citric acid cycle and is deviated to ketone synthesis, usually due to a relative lack of glucogenic substrate. Despite the lowest body weight and body condition in Langano fish, they had the lowest 3OHC4:C2 ratio, which means that their poorer performance was not due to a relative lack of glucogenic substrate rather than an overall insufficient nutrient supply that reduced their metabolic rate, as witnessed by the low C2:C0 ratio.

This relative lack of glucogenic supply to the citric acid cycle, therefore, does not seem to come from a relative lack of glucogenic substrate but can point to a deficiency of specific micronutrients needed to support the conversion to succinyl CoA, such as magnesium. We indeed found a lower magnesium status in fish from Lake Langano^37^^.^ The present metabolite analysis thus points to further routes of investigation to identify the underlying causes of poor development in fish.

The largest fish were found in Lake Ziway, yet with less body fat. Based on the intermediate values of the C2:C0 ratio, their metabolic rate was not as high as in the smaller Gilgel Gibe fish. This can be explained by a higher growth efficiency: because fat is highly energetic per unit of weight, lean growth comes with a higher food utilization efficiency: less food will be needed for the same amount of growth when that growth is leaner (protein and water) compared with fat deposition. Remarkably, Lake Ziway fish had the lowest C4DC: C2 ratio, suggesting that relatively less protein was needed to support the generation of energy. A possible, but so far not investigated route, is the use of chitin as an energy source. Many aquatic organisms such as crustaceans and insects have an exoskeleton containing chitin^38–40^. It has been demonstrated that many fish species secrete endogenous chitinase in their gut^41–43^, but very little information is available on the metabolic fate of chitin that is enzymatically degraded. Chitin mainly consists of N-alpha-acetyl glucosamine units, which after absorption, should split in an acetyl group that can provide acetyl CoA and glucosamine that can easily convert to glucose. That would mean that fish would still have another glucogenic substrate apart from amino acids and eventually propionate from fermentation, but, as said, this is still uncovered. Yet, it could explain the high lean growth of Lake Ziway fish. The gut content analysis of Nile tilapia from Lake Ziway showed a higher proportion of zooplankton (crustacean and rotifer) and aquatic insects^29^. Crustaceans and aquatic insects are the main chitin source in the aquatic environment^44^. Thus, a larger size of Lake Ziway fish, but lower fat content (leaner) compared to smaller size Gilgel Gibe fish is possible, Lake Ziway fish would use chitin as an alternative glucogenic substrate than an amino acid.

In conclusion, the nutrition-related blood metabolite ratios in our study explained the habitat-dependent differences in the growth of Nile tilapia. Therefore, we provide a method to explore the underlying causes of growth differences in fish.

## Material and methods

### Animal ethics statement

This study was checked and given approval by Jimma University, College of Natural Sciences Research and Ethical Review Board Committee (Jimma, Ethiopia). The committee has followed the standard procedures written on Article 6 (methods of killing) and Article 9 (Animals are taken from the wild), of directive 2010/63/EU of the European parliament and of the council of 22 September 2010 on the protection of animals used for scientific purposes and certified this study with the letter referenced (Ref. No: RPG/165/2019). This study was carried out in compliance with the ARRIVE guidelines.

### Description of the three lakes in this study

Fish samples from the three lakes were used in this study, and a summary of limnological information is given in (Table 5)^37,45^. Lake Ziway is located in the central Rift Valley zone of Ethiopia. It has open water and flat swampy margins on all sides except the south and southwest. It fills a depression at an elevation of about 1636 m above sea level. Meki and Ketar Rivers are the main tributaries of the lake. The lake is the shallowest lake in the country and drains into Lake Abiyata via Bulbula River. It is the third-largest freshwater lake in the country. It has a surface area of 434 km^2^ and has five islands: Gelila, Debre Sina, Tulu Gudo, Tsedecha, and Fundro. The climate of the lake basin is dry to sub humid. The lowland area surrounding the lake is arid or semiarid, and the highlands are sub dry humid to humid.

**Table 5:** Geographic description of the three Ethiopian lakes in this study.

Parameters	GG	LZ	LL
Latitude	7°42′53″–7°55′580″N	7°20′54″–8°25′56″N	7°03′0″–7°04″2″N
Longitude	37°11′53″–37°20′33″E	38°13′02–9°24′01″ E	38°04′0″–38°04′9″E
AL (m) asl	1640	1636	1582
SA (km^2^)	62	434	241
CA (km^2^)	4200	7025	1600
MD (m)	17.6	2.5	17
DC (km)	283, south-west	165, south	200, south

GG: Gilgel Gibe; LZ: Lake Ziway; LL: Lake Langano; CA = catchment area; SA = surface area; DC = distance from the capital; AL = altitude; MD = mean depth.

Lake Langano is one of the northern Rift Valley lakes of Ethiopia. It is situated at an altitude of 1582 m and has a surface area of 241 km^2^ and a mean depth of 17 m. The lake is highly turbid, and the water is usually reddish-brown; it is known by its nickname “Golden Lake”. It is mainly fed by runoff and hot springs. The lake water discharges into Lake Abijata via Hora Kello River. The climate is rainy between June and September, followed by a dry season from November to February.

Gilgel Gibe reservoir is located 283 km southwest of the capital and 70 km northeast of Jimma town. The dam is constructed at an altitude of 1640 m above sea level. Its maximum and minimum water levels during wet and dry seasons are 1671 and 1653 m above sea level, respectively. Its depth ranges between 2 and 35 m, with a mean depth of about 17.6 m, and it covers a total surface area of 62 km^2^. It is bordered by three districts (woredas): Omo-Nada, Kersa, and Tiro-Afeta. The area has a subhumid, warm to hot climate and receives between 1300 and 1800 mm of annual rainfall; it has a mean annual temperature of 23.7 °C. In addition to hydropower generation, the reservoir plays an important role for the local community as a fishery resource.

### Fish sample collection and euthanasia

Nile tilapia samples were collected during dry season (November, 2018) from three the Ethiopian lakes (Gilgel Gibe, Ziway, and Langano) (Table 5). For this particular study, Nile tilapia was selected based on its relevance to commercial fishing in the country and the fish consumption by the local population. A total of sixty (N = 60) Nile tilapia samples were collected comprising twenty (n = 20) fish from each lake caught using the same gillnets. Immediately, the fish were euthanized by pithing the brain^46^ for blood collection.

Then, the samples were thoroughly washed with tap water to remove any adhering contaminants and then transported using an icebox to the laboratory (samples from Gilgel Gibe reservoir were transported to Jimma University Zoological Sciences whereas samples from Lake Ziway and Langano were transported to Batu Fisheries and Other Living Aquatic Resource Research Center). Upon arrival, all fish were measured for their total body length (standard + fork length) and total weight to the nearest 0.10 g and 0.01 cm respectively. Afterward, the samples were re-washed thoroughly with potable water, then dissected (using plastic tools) and targeted tissues (muscle, gills, skin, gut, and liver) were collected. Macronutrient composition; dry matter, crude protein, and crude fat content of all targeted tissues were determined.

### Water quality parameters of the three lakes

On-site physicochemical water quality measurements of the three lakes were measured. Day-time dissolved oxygen, water temperature, electrical conductivity, and pH were measured using a multi-probe meter (HQ40D Single-Input Multiparameter Digital Meter; Hach Company, Loveland, CO, USA). In addition, the concentration of nitrate and ammonia were also measured on-site using a Palintest multiparameter (Photometer 7500; Tyne and Wear, UK, NE11 0NS) (Table 1).

### Blood sampling and analysis of acylcarnitine

Blood samples were drawn from the caudal vein^47^ by puncturing with a 1 mL syringe (Becton Dickinson S.A., Madrid, Spain) and a 26-G needle (Becton Dickinson, Drogheda, Ireland) rinsed with heparin (LEO Pharma, Ballerup, Denmark). Blood collection from each fish lasted less than 3 min to avoid cortisol increase^48^ due to manipulation upon sampling. Consequently, the blood samples were blotted and dried on filter paper (Whatman903, Cardiff, CF147YT, UK). The dried blood spot (DBS) samples were shipped to Ghent University, Belgium, for further analyses. Finally, the acylcarnitine and free amino acid profile of the DBS was determined using quantitative electrospray tandem mass spectrometry following^49,50^ at the Laboratory for Metabolic Diseases at Ghent University Hospital. Metabolite ratios were calculated from relevant biochemical pathways that could identify relative changes in nutrient metabolism. The metabolite ratios used in this study were as followed;—Acetyl : Free carnitine (C2:C0); Propionyl:Acetyl (C3 : C2);3-hydroxybutryl : acetyl(3OH-C4:C2);Methylmalonyl : Propionyl(C4DC:C3); Methylmalonyl : Valine + Methionine(C4DC : vet. + Met.);Malonyl : Acetyl(C3-DC : C2); Valine : Leucine (Vet : Leu) and sum of 3- hydroxyl long-chain fatty acids : sum of Long-chain fatty acid (3-OH LCFA : LCFA).

### Macronutrient analysis (proximate analysis):

The proximate composition of all targeted tissues of Nile tilapia was determined by conventional methods described by the Association of Official Analytical Chemists^49^ for crude fat) and crude protein.

### Crude fat determination

Crude fat was analyzed using the ether extract method^51^. A 2 g dried sample was inserted into a porous thimble allowing rapid flow petroleum ether. The sample was wrapped in filter paper, placed into the thimble, and covered with glass wool. Anhydrous ether was placed into a weighed boiling flask, which, together with the Soxhlet flask and condenser, was assembled into the Soxhlet apparatus. Crude fat was extracted into a Soxhlet extractor for 6 h, by the heating solvent in the boiling flask. The boiling flask with extracted fat was dried in an air oven at 100 °C for 30 min, cooled in a desiccator, and weighed. Finally, the percentage of crude fat was computed following.

### Crude protein determination

Crude protein was determined by an automatic Kjeldahl analyser methods^52^, using Velp Scientifica (UDK 159, F30200150). A VELP DK Series Digestion Unit and a VELP UDK 159 automatic digestion and titration system were used subsequently. Every sample flask was prepared by adding 2 catalysts for digestion. This included 1 g of the dried sample, 0.2 g CuSO4, and 7 g of K_2_SO4. For the actual digestion, 20 ml of H_2_SO4 was added. The program was set at 30 min at 250 °C, 30 min at 350 °C, and 1 h at 420 °C. Ultimately the samples were cooled down to 50 °C.

Then, distillation took place by adding distilled water followed by adding 10 ml and 7 ml of indicators bromocresol green and methyl red respectively. The nitrogen was separated from the digested mixture by steam distilling with the UDK 159 to extract ammonia from the alkaline solution. Sodium hydroxide (35%) was added to raise the pH and convert solid NH_4_^+^ into gaseous NH3. The distilled nitrogen was then trapped by adding boric acid.

For the final colorimetric titration, hydrochloric acid was added to react with the ammonia and the indicators. The volume of titrant that was needed to reach the endpoint allows to automatically calculate the amount of nitrogen, expressed as a percentage of nitrogen or display the percentage of protein directly.

### Statistical analyses

All data were evaluated for normality. The crude protein and crude fat content of tissues were analyzed by repeated measure analysis of variance within tissues as within-subject variable and lake as between-subject variable. One-way analysis of variance was used to analyze the ratios of selected acylcarnitines and free amino acids. For both analyses, a subsequent post-hoc comparison using Tukey’s test was performed. All analyses were done using the statistical package of SPSS version 26.0. The significance was treated at *P* < 0.05 confidence level.

## Data Availability

The dataset generated during the current study are available from corresponding author on reasonable request.
